# Introducing multi-component cardiovascular health screening into existing Abdominal Aortic Aneurysm (AAA) screening programmes in the UK: a qualitative study of programme staff views

**DOI:** 10.1186/s12913-022-07975-7

**Published:** 2022-04-28

**Authors:** Maria Zubair, Matthew J. Bown, Natalie Armstrong

**Affiliations:** 1 Department of Health Sciences, George Davies Centre, University of Leicester, University Road, Leicester, LE1 7RH UK; 2 Department of Cardiovascular Sciences and NIHR Leicester Biomedical Research Centre, University of Leicester, University Road, Leicester, LE1 7RH UK

**Keywords:** Cardiovascular screening, Hypertension, Peripheral arterial disease, Programme delivery, Screening programme, Staff views, Qualitative

## Abstract

**Background:**

Cardiovascular disease is a major contributor to poor health in the UK and the leading cause of death in England. Peripheral arterial disease and high blood pressure are conditions that identify individuals at high cardiovascular disease risk, likely to benefit from cardiovascular risk management. Both conditions remain considerably underdiagnosed and untreated. The National Health Service abdominal aortic aneurysm (AAA) screening programmes represent an opportunity to screen for these conditions with potentially minimal additional effort or cost. We explored AAA screening programme staff views on the proposed introduction of such additional screening within AAA screening.

**Methods:**

Nine focus groups and seven follow-on interviews were undertaken with 38 AAA screening staff. Our study methods were oriented broadly towards a grounded theory methodology, and data were analysed using thematic analysis.

**Results:**

Three themes were identified: (i) ‘*Perceptions of patient experience and health-related outcomes*’, (ii) *‘Opportunities and challenges for programme staff’*, and (iii) *‘Maintaining and improving programme standards’*. Staff talked about the high uptake of AAA screening, staff experience and skills in their role, and the programme’s high quality standards as both opportunities and potential challenges linked to the proposed additions to AAA screening. While positive about the potential to improve patients’ health outcomes, participants had questions about the practicalities of incorporating additional procedures within their time- and resource-constrained context, and how this may reconfigure work processes, roles and relationships.

**Conclusions:**

The proposed additions to the programme require taking staff’s views into account. Key areas that need to be addressed relate to ensuring follow-up support for patients, clarity around staff responsibilities, and availability of sufficient resources for the programme.

## Background

Cardiovascular disease (CVD) is a major contributor to poor health, and one of the leading causes of death in the UK. In 2018, ischaemic heart diseases alone represented the largest number of deaths in men (40,214) in the UK, and second largest among women (23,662) [1]. The NHS Long Term Plan identifies early detection and treatment of CVD as a priority for the National Health Service (NHS) [2]. However, the systematic identification of individuals at high risk of future CVD is problematic. In the UK, the main NHS preventative strategy for the management of cardiovascular risk is the NHS Health Checks system. Attendance for these Health Checks is regarded as sub-optimal [3, 4], with the national average uptake for 2014–2019 being 48% (which includes checks that are done opportunistically) [4].

With regards to prevention of CVD, detection of peripheral arterial disease (PAD) and high blood pressure (BP) offers an important means of identifying individuals at high CVD risk in whom preventative cardiovascular risk management will be beneficial in accordance with the European Society of Cardiology Guidelines [5]. The National Institute for Health and Care Excellence recommends cardiovascular risk management for individuals with a 10-year CVD risk of greater than 10% [6]. Men with symptomatic PAD have a 10-year risk of over 25% for major adverse cardiovascular events [7]. Moreover, patients with asymptomatic PAD have similar long-term cardiovascular risk [8], with studies demonstrating 5-year cardiovascular risks of around 20% for asymptomatic individuals [9, 10]. Similarly to PAD, high BP is also a strong independent risk factor for future cardiovascular events [11, 12], with individuals with high BP having a lifetime risk of 63.3% for overall CVD compared with 46.1% for those with normal BP, and developing CVD five years earlier [12].

Despite the associated risks of CVD, both PAD and high BP are considerably underdiagnosed and untreated [13–17]. Empirical evidence suggests that the true population prevalence of PAD is between 13 and 18% [18–20], yet UK primary care data records a much lower prevalence of diagnosed disease at around 3% [13]. Similarly, over 7% of 40–74 year old men in the UK without a history of CVD have been found to have untreated high BP, with this prevalence particularly high among those aged over 55 years [15]. Within this context, the NHS abdominal aortic aneurysm (AAA) screening programmes, with their high uptake (over 81% in England during 2018/19 [21]), represent an opportunity to screen for possible CVD through the addition of PAD and high BP screening to the programme. It is important to note here that the detection of AAA itself can also identify individuals at a higher CVD risk than the general population [22, 23]. Published evidence relating to CVD among individuals with a small AAA has, for example, revealed a prevalence of ischaemic heart disease, myocardial infarction, heart failure, and stroke within this population at a rate of 44.9%, 26.8%, 4.4%, and 14.0% respectively [23]. The addition of PAD and high BP screening to the existing AAA screening provision, however, offers the opportunity to additionally identify individuals at high CVD risk who may not necessarily have an AAA.

An expansion in the scope of screening programmes, as proposed here, has been successfully trialled previously in Denmark where the multifaceted cardiovascular screening incorporated screening for aneurysms, PAD, uncontrolled hypertension, atrial fibrillation, severe hyperlipidaemia, and diabetes mellitus [24]. Furthermore, the recent Danish Viborg Vascular (VIVA) trial has involved screening specifically for PAD and high BP together with AAA screening. Evidence from this trial demonstrated a significant reduction in all-cause mortality among men invited for screening, with prevalence of PAD, high BP and AAA being 10.5%, 10.9% and 3.6% respectively, and the effect on overall mortality being a hazard ratio of 0.93 after a median follow-up of 4.4 years [25]. Hence, while a multi-component cardiovascular screening programme can potentially target a range of different cardiovascular risk factors, the rationale for adding PAD and BP screening particularly to AAA screening is based on the VIVA trial, which has provided favourable evidence, with adequate follow-up, particularly on the effectiveness of combined AAA, PAD and high BP screening.

The established AAA screening infrastructure in the UK may allow for delivery of multi-component AAA, PAD and high BP screening with potentially minimal additional effort or cost. The introduction of PAD and high BP screening within the existing AAA screening programmes may fit well with the existing model of AAA screening organisation and delivery in the UK, and be undertaken by existing screening staff. However, how well this works in practice needs to be investigated. Therefore, as part of a wider programme of work to determine the feasibility as well as effectiveness of such additional screening, we explored NHS AAA screening programme staff’s views regarding the proposed introduction of the additional components of PAD and high BP screening within existing AAA screening programmes. Within the proposed model, while screening for PAD and high BP would be undertaken by AAA screening staff, those screening positive for these would receive follow-up CVD risk management through general practice rather than through the AAA screening programme.

## Methods

This qualitative study involving focus groups and follow-on semi-structured interviews formed part of the National Institute for Health Research (NIHR) funded Peripheral arterial disease, High blood pressure and Aneurysm Screening Trial (PHAST), which aims to look at the effectiveness, cost-effectiveness, feasibility and acceptability of adding screening for PAD and high BP to the NHS AAA screening programmes. As part of the PHAST programme, NHS professionals across England with experience of working in AAA screening were invited to attend one-day workshops at the University of Leicester during April–May 2021. After an introduction to the PHAST programme, and hands-on testing and reviewing of a range of suitable screening devices, focus groups were undertaken with the attendees who provided informed written consent to take part.

Participants were approached for the workshops through an email invitation distributed to all NHS AAA screening services across England. Recruitment was enhanced through wider advertisement of the workshops via Twitter and professional networks of the PHAST research team. The target sample size was a minimum of twenty-five participants. In total, thirty-nine AAA screening programme staff attended the workshops, and all but one took part in the subsequent focus groups (one could not stay due to time constraints). Focus group participants included staff working within twelve different NHS AAA screening services across England, representing a range of screening sites spread over both urban and rural locations. These participants worked in a variety of roles, including as AAA screening technicians, programme managers, clinical scientists/ clinical skills trainers, and AAA specialist nurses.

The aim of the focus groups was to explore participants’ views regarding the proposed introduction of PAD and high BP screening within the existing NHS AAA screening programmes, and particularly what they considered or anticipated to be the possible challenges and opportunities this may present. The focus groups were guided by a semi-structured topic guide, developed specifically for this study and informed by research team members’ expertise in the areas of cardiovascular health screening and healthcare organisation and delivery. It was used flexibly to accommodate relevant topics emerging in the group discussions. In exploring participants’ views regarding the proposed additions to the AAA screening programmes, the topic guide also covered relevant areas such as: the practical utility of the various PAD and high BP screening techniques/devices within the AAA screening context; delivery of screening results; training and support needs of programme staff; and optimisation of screening attendance.

Nine focus groups were conducted, lasting 20–45 min. These were supplemented with seven follow-on interviews, allowing more in-depth exploration of participants’ views on an individual basis. The interviews also provided an opportunity to check the researcher’s interpretations of the focus group data and receive participants’ feedback on the study’s emergent findings. These interviews, carried out by telephone, lasted for approximately 45 min and involved participants who had consented to follow-on interviews. At the participants’ request, one interview involved two participants who worked together. All data collection (face-to-face focus groups and telephone interviews) was undertaken in quiet rooms with no other persons present besides the participants and researchers.

The focus groups and interviews were conducted by MZ – a female post-doctoral researcher with expertise in qualitative health research. Four focus groups were co-facilitated by MJB – a male clinical researcher and study lead, who is a professor and consultant vascular surgeon. Through his clinical role, MJB had previously engaged with some of the participants at professional meetings where he had talked about the PHAST programme prior to study commencement.

All focus groups and interviews were audio-recorded for transcription. Brief field notes capturing the researchers’ reflections and contextual details were also made. Quality and accuracy of the transcribed data was checked against the audio-recordings, rather than by returning transcripts to participants, and data collection continued until the point of reaching thematic saturation. Our study methods were oriented broadly towards a grounded theory methodology [26]. The data were analysed using thematic analysis [27] and an inductive approach to coding. The analysis began with an initial familiarisation with the data, followed by a first stage of open coding by MZ that led to the generation of an extensive list of initial codes which were reviewed and assessed by NA and then applied systematically across the focus group and interview data. All data management and coding was undertaken using Microsoft Excel. The codes were then grouped and regrouped together and organised into potential themes that appeared to be emerging from the data. These themes were discussed and assessed within the research team in terms of what these incorporated, then reviewed across the dataset in an ongoing process of theme refinement and adjustment until clear definitions and names were generated for each theme.

Ethical approval for the study was obtained from the University of Leicester Ethics Sub-Committee of Medicine and Biological Sciences (ref: 26,165).

## Results

Three main themes emerged from the data: (i) *‘Perceptions of patient experience and health-related outcomes*’, (ii) *‘Opportunities and challenges for programme staff’*, and (iii) *‘Maintaining and improving programme standards’*. We discuss these themes with example short data extracts. Further supporting data are shown in Tables 1, 2 and 3.

**Table 1:** Subthemes of theme I, with illustrative quotes

Theme I – Perceptions of patient experience and health-related outcomes	
**Sub-themes**	**Quotes**
1a) An opportunity to improve health-related outcomes 1b) Positive health outcomes dependent on adequate follow-up 1c) PAD + BP + AAA screening’s impact on patient experience	*“I think it’s the fact that, you know, we’re attracting essentially every sixty-five-year-old *gentleman nationally, aren’t we, so – it’s almost like a gift ….. to pick up something else is *got to be good ……”* (AAA Screening Programme staff, Focus group 7) *“….. people might actually be more inclined to have it done as part of AAA screening than going for like a health check at the GP surgery ….. and it's just about preventing people um from, you know, coming in as an emergency or being referred into the hospital setting ….. until they need it really ….."* (AAA Screening Programme manager & formerly screening technician, Interview 7) "*Well depending on um if there is a (unclear) for the GP to act on, or a protocol to act on, then you can expect a bit of a favourable action. Sometimes you do all this – send it to them, and it's ignored or neglected, then it seems pointless, unless it's something to be actioned, or if they have a guideline to say that if this is a possibility, or this is this, this is what we're supposed to do, then we can expect a bit of a benefit or a good outcome of our screening ……*" (AAA specialist nurse & screening technician, Interview 5) *"….. I don't think that every man found to have PAD will go and book an appointment with their GP ….. I think it would be more beneficial for the patient to be booked an appointment – even if it was just a one-off, with the vascular nurse ….. They would be able to explain to them exactly why it's important they make the changes that they do make ….. because some GP surgeries would be better than others if the man does even attend …..*" (AAA Screening Programme manager & formerly screening technician, Interview 7) *"And I see it as the more you're doing for one quick appointment ….. they don't have to come back again ….. they're only coming one appointment, they're not having two appointments, even if it's twenty minutes or half an hour – it's not like they'll have to be there for three hours, and have to pay like loads for the parking ….."* (AAA Screening Programme staff, Focus group 6) *"I'm on about to make the appointment as efficient as possible for the patient – we need something that you just literally clip on the toe or clip on the arm – or put on the arm, and there you go – there's your result ….. The easier things are to administer, you don't have the stresses and anxieties of um you know – difficulties being experienced while you're undertaking those tests ….."* (AAA Screening Programme staff, Focus group 8)

**Table 2:** Subthemes of theme II, with illustrative quotes

Theme II – Opportunities and challenges for programme staff	
**Sub-themes**	**Quotes**
2a) Positive extension and growth in screeners’ role 2b) Incorporating additional procedures in a resource-constrained context 2c) Reconfiguration of roles, responsibilities and relationships	*“….. it is a role that doesn't go anywhere ….. With the introduction of this health screen, it has transferability into other screening programmes ….. like I said, it isn't a role where they can extend upwards, past the one before ….. as this will be an additional element in their role that they'll be screening two conditions um rather than one ….. so I think overall – yes, it is a positive thing. With the detection rate for triple A is declining, I think there needs to be an additional um function to the role as well – to make the – to ensure the continued viability of the screening programme …..”* (AAA Screening Programme administration manager, Interview 1) *“So, in some programmes, technicians become bored because of the repetitiveness of the triple A, and if you introduce um PAD and BP screening, then that will introduce different element to their role ….."* (AAA Screening Programme staff, Focus group 8) *“….. additional time to remove extra clothing ….. I mean at the moment all we do is, we just ask gentlemen to lift their upper clothes up – that's all we have to do, so if you're incorporating 'You need to remove your shoes and socks, then you know, that's a whole different – a whole different game.”* (AAA Screening Programme staff, Focus group 6) *"I think a lot of our screening programmes did ten minutes with two technicians – that obviously increased to initial Covid-secure, sort of, um appointment. Um so it's gonna be interesting ….. will be interesting to see if programmes can actually do that.”* (AAA Screening Programme staff, Focus group 5) *"….. in how we work – it would almost, if we were doing the trial – that it would almost be better to have a separate clinic, specifically for that, so it wouldn't – 'cause obviously we've all got catch-up to do after Covid, you know – we're way behind in our numbers. So like, in the next few years, you know – we've got pressure already there from – from – 'cause we've got 9,000 people to scan a year ….."* (AAA Screening Programme staff, Focus group 2) *"You're there to screen, and the results ….. I think as well, when it comes to giving results ….. because, you know, managing the blood pressure is really primary care, isn't it. It's down to the GP. Um you know, how much do you say to these people ….. It has to be very clearly defined ….. it's quite clear (within AAA screeening programme), isn't it – you're also um getting them an appointment with the nurse. You are actually responsible for the next stages with the nurse, whereas with this (i.e. PAD* + *BP screening) ….. you're sending them up to the GP, which is really mixed – everything is different ….."* (AAA Screening Programme staff, Focus group 2) *"The other thing would be the actual – because it's GP that decides whether they – what they decide to do or not ….. With the aneurysm screening ….. two weeks on, or whatever the timescales are, you're likely to have an operation – not definitely – not guaranteed, but it's a possibility. Actually, if you go to GP, he's too busy to do – or doesn't think the patient has got peripheral arterial disease, or something like that – very – quite variable."* (AAA Screening Programme staff, Focus group 5)

**Table 3:** Subthemes of theme III, with illustrative quotes

Theme III – Maintaining and improving programme standards	
**Sub-themes**	**Quotes**
3a) Maintaining AAA programme standards 3b) An opportunity to improve service-delivery	*"As long as you've got time for it – not to impact on the quality of the triple A screening ….. We've got it (i.e. AAA screening) good, and how we do that, and you know – and the timing we've got, you know, is appropriate for that ….."* (AAA Screening Programme staff, Focus group 2) *"It's always standards, isn't it – you tend to sort of set up your achievable and your acceptable standards – your thresholds um – how they're gonna be monitored. Um I think it is your appointment times, um practicalities, equipment – buying the equipment, whose gonna buy the equipment – whose going to actually um replace the equipment ….."* (AAA Screening Programme staff, Focus group 5) *"If you've got lots of false positives or false negatives then it undermines the whole screening programme ….. so you'd want to know who would be looking at what we're doing, and whether or not it's actually being checked to make sure ….. we need a QA (Quality Assurance) ….."* (AAA Screening Programme staff, Focus group 6) *"Could this be a way of getting that letter into – *via* what's called the Patient Knows Best app, so you can opt into it so you get copies of letters that go to your GP that's about you – whereas the triple A isn't in there – whereas if you're getting funding, if you've got this then the patient could have the letter ….. I mean, you give them the result on the day, but a lot of the time they don't take in a verbal result ….. For certain people, they like to have written results ….. So if you're thinking of electronically doing a lot of stuff, then maybe we can do this ….."* (AAA Screening Programme staff, Focus group 6) *"I think it would benefit us ….. it's an incentive for the GPs – whereas GPs I've found are very dismissive of us when we go. I mean, we do pay for the room, but we're more of an inconvenience at the minute – whereas if GPs are on board with this, and we're doing the service in there ….. So hopefully they'll play their part a bit better than what they do now ….. I think they're gonna be more on board with it and they'll probably welcome us to come around – 'cause also then, we're doing it, they're not having to train up a lot of staff to do it for them ….. it will open, I think, more doors for us ….. I think, this will benefit triple A as well ….. we feel like we're in the way a little bit although we try not to ….. we just think this will be an incentive for them."* (AAA Screening Programme staff, Focus group 6)

### Theme I: Perceptions of patient experience and health-related outcomes

Screening programme staff anticipated that the proposed multi-component screening programme would most likely have an impact on both patient experience and health-related outcomes. Their discussions relating to how the proposed programme may affect patients comprised three sub-themes: (i) An opportunity to improve health-related outcomes, (ii) positive health outcomes dependent on adequate follow-up, and (iii) PAD + BP + AAA screening’s impact on patient experience.

#### Sub-theme 1a: An opportunity to improve health-related outcomes

Participants’ views regarding the proposed introduction of PAD and high BP screening into the AAA programme were overwhelmingly positive with respect to it being an opportunity to improve health-related outcomes for attendees. Screening staff unanimously saw PAD and high BP screening as something beneficial that would enable “*picking up on conditions that wouldn't be picked up on*” otherwise, and the AAA screening programme as a particularly effective means of ensuring good coverage because “*you've got…this whole sixty-five-year-old group of men coming to you, so the opportunity to make sure you don't miss anybody*” (Focus group 7). Alongside an emphasis on how AAA screening’s high coverage was helpful, participants suggested the multi-component aspect of the proposed PAD + BP + AAA screening is likely to also receive a positive response by attendees who “*will appreciate it, because they'll see more of a health check-up, and…they'll be quite keen to come*” (Focus group 6).

#### Sub-theme 1b: Positive health outcomes dependent on adequate follow-up

Participants highlighted that the positive health outcomes from PAD and high BP screening were largely contingent upon adequate follow-up in terms of provision of cardiovascular risk management support, suggesting that an important question to address was: "*In case if the GP [i.e. General Practitioner] doesn't action it, what back-up plans do we have?*" (Focus group 4). The participants thus raised the potential for inaction on the part of GPs, and noted that PAD and high BP screening could introduce “*a lot of additional work*” only to find that “*nothing happens, 'cause we're [i.e. screening programme staff] never gonna follow these gentlemen up*” (Focus group 2). Participants suggested things they would like to see put in place, such as clear, actionable guidelines and protocols for GPs to follow and initial input from AAA screening vascular nurses.

#### *Sub-theme 1c: PAD* + *BP* + *AAA screening’s impact on patient experience*

Participants envisaged PAD + BP + AAA screening as having the potential to be experienced favourably in so far as it allows getting more tests to be done within one appointment. Effective management of time “*to make the appointment as efficient as possible for the patient*” (Focus group 8) was hence identified as critical to ensuring that the addition of PAD and high BP screening did not impact negatively on patient experience. Emphasising particularly that AAA screening requires a simple ultrasound scan, some participants characterised PAD and BP screening processes as potentially more complicated, and suggested needing to find tests that are easy to administer as they reasoned that “*keeping it simple, reduces the length of time patients are there, so they're not getting anxious and distressed*” (Focus group 8). Furthermore, participants highlighted their own interactions with screening attendees as contributing to positive patient experience, and suggested this aspect needed to be retained within any reworking of future screening schedules since “*you can't just throw them out – you have to talk to them 'cause we might be the only people they see all day*” (Focus group 7).

### Theme II: Opportunities and challenges for programme staff

The proposed screening programme was viewed by AAA screening programme staff as having the potential to present both new opportunities as well as challenges for them. Three main sub-themes emerged from the data in relation to the potential opportunities and challenges for screening programme staff: (i) Positive extension and growth in screeners’ role, (ii) incorporating additional procedures in a resource-constrained context, and (iii) reconfiguration of roles, responsibilities and relationships.

#### Sub-theme 2a: Positive extension and growth in screeners’ role

Participants viewed the proposed PAD + BP + AAA screening as a good opportunity for developing and diversifying the screening technician role. It was observed that AAA screening technicians have “*a fairly limited role*” which “*can get a bit monotonous*” and “*hasn't really got a career path to go on from that*” (Interview 1). AAA Screening Programme Managers further highlighted that this has also meant that “*it's quite a hard role to recruit to*”. Within this context, the introduction of PAD and high BP screening was regarded as “*a hugely positive step to take*” (Interview 1). The benefits for screening technicians were identified as not merely including a diversification and greater transferability in their skills, but also the development of a more fulfilling work role – one which provides “*extra development*” and something to “*challenge your brain*” (Focus group 5), along with providing the enthusiasm “*to want to come in to work, to be enthused to impact patients – to benefit patients*” (Focus group 8).

#### Sub-theme 2b: Incorporating additional procedures in a resource-constrained context

PAD and high BP screening were characterised by many participants as involving additional, longer processes to those within AAA screening. These participants stated that “*from start to finish, it is a separate screening*” (Focus group 1) and “*a whole different game*” to AAA screening (Focus group 6). With respect to incorporation within the AAA screening programme, participants emphasised that while “*the premise of it is brilliant*” they were less clear about “*how it's gonna work out – in practice*” (Focus group 3). Participants particularly queried how the screening would be introduced, suggesting that there would “*need to be a lot of thought into the actual day-to-day operation of it*” (Focus group 8). Describing the time and resource constraints experienced within their existing AAA screening context, and the pressure “*to get through the cohort*” (Focus group 1), participants identified the proposed additions as a potential challenge whereby they may be required to manage the number of patients they “*need to see every year*” against “*the time it will now take, adding on this additional test*” (Focus group 5). Participants noted, however, that these challenges may be addressed through provision of sufficient resources, suggesting “*if that's costed and staffed…that is not insurmountable*" (Focus group 5).

#### Sub-theme 2c: Reconfiguration of roles, responsibilities and relationships

Participants envisaged that the additional screening would inevitably reconfigure their roles and responsibilities together with work relationships across organisational boundaries. Screening technicians, in particular, observed: “*GPs I've found are very dismissive of us when we go…we're more of an inconvenience at the minute*” (Focus group 6). These participants suggested that undertaking the additional screening may “*garner more respect for*” their role (Focus group 3). While expressing enthusiasm for a diversification in their role, participants emphasised: “*we need a bit more of clarity in it, then we'll know where we are…what our responsibilities are*” (Focus group 4). The screening additions were hence not perceived by all participants as necessarily unambiguous extensions to their screening roles but ones which potentially re-defined and could complicate the boundaries and remit of their existing role and its associated responsibilities, as one participant explained: “*as standard practice for a screening technician, you're not there to diagnose…you're not diagnosing for aneurysm screening, but you're almost being asked to diagnose for peripheral arterial disease…you'd gotta be careful not to step on the toes of the GP*” (Focus group 2).

### Theme III: Maintaining and improving programme standards

An important concern of the screening programme staff with regards to the introduction of the additional screening related to the maintenance of, and improvement in, screening programme standards. Screening programme staff’s views regarding the opportunities and challenges presented by the additional screening with respect to the AAA screening programme standards comprised two sub-themes: (i) Maintaining AAA programme standards, and (ii) an opportunity to improve service delivery.

#### Sub-theme 3a: Maintaining AAA programme standards

Participants noted the declining AAA detection rates, observing that “*at some point in the future there is likely to be some diversification within AAA screening*”, and in this respect the incorporation of additional screening “*futureproofs triple A screening*” (Focus group 8) and ensures “*the continued viability of the screening programme*” (Interview 1). While looking upon this prospect favourably, participants nonetheless highlighted the high standards of quality maintenance and service delivery within the AAA programme stating that they “*would hate for that to be…put in any sort of a detriment"* as a result of the proposed additions (Focus group 2). Maintaining the existing programme standards was hence identified as an important consideration. These standards were invariably described as relating to “*the level of care”* provided to patients (Focus group 7); safety, hygiene and infection control; appointment timings; accuracy of test results; adequate follow-up etc., and included formalised processes and assessments. Participants stated: “*we're assessed regularly to make sure we're maintaining the standards…we need the equivalent (for PAD and high BP screening)*” (Focus group 6).

#### Sub-theme 3b: An opportunity to improve service delivery

In addition to emphasising the high standards of service delivery within the existing AAA programme, participants also saw some potential for improvement through the addition of the proposed screening. Two areas that participants identified where they saw an opportunity arising for improvement related to the provision of written results to screening attendees who do not screen positive for an aneurysm but are then “*ringing up again wanting to book, and they've already had it, but they've got no memory of it*”, and also “*open(ing) up (screening) locations…get(ting) a wider group of surgeries on board, and spread(ing) the message a bit more*” (Focus group 6). In both these cases, participants suggested that the new processes, resources and/or roles and relationships developing as part of the additional screening could “*help improve triple A as well*” (Focus group 6).

## Discussion

The study findings suggest that AAA screening programme staff viewed the addition of PAD and high BP screening to the NHS AAA screening programmes as a positive development overall. The proposed screening additions were not only perceived as having good potential for improving patients’ health-related outcomes, but also as offering possible benefits at the same time for screening staff and the AAA screening programme itself. Participants, however, identified that the benefits of incorporating the additional screening would be largely contingent upon a number of appropriate systems, structures and resources being put in place. These related, in particular, to the potential challenges anticipated around three key areas that needed to be addressed.

Firstly, a potential challenge that was highlighted related to ensuring the provision of adequate follow-up support for cardiovascular risk management to persons who screen positive for PAD and/or high BP. This entailed a need to also identify who really is best placed to provide such follow-up support. While the evidence on the low uptake of the NHS Health Check for cardiovascular risk assessment and management (provided mainly through general practice) is unequivocal [3, 28, 29], it has also been recognised that the accessibility barriers to CVD screening may also be linked specifically with how the general practice context itself is organised, perceived and/or experienced by different patient groups [30–32]. In line with such evidence, screening programme staff expressed their uncertainty around the adequacy of follow-up of screened positive patients beyond the AAA screening programme context. Raising the potential for inaction on the part of general practice, participants suggested the need to put appropriate processes and structures in place to ensure a consistent and adequate follow-up of screen positive patients.

Secondly, participants anticipated the likelihood of their roles and responsibilities being expanded with the additional screening. While such an expansion would require the provision of appropriate training and support for screening staff, this study’s findings reveal some appetite amongst screening staff and managers to take advantage of this potential opportunity for extra development and learning. Participants’ responses, however, emphasised that the remit and boundaries of their specific roles and responsibilities needed to be clearly defined as these were expanded – particularly vis a vis GPs and other primary care health professionals, working across organisational boundaries, but responsible for similar cardiovascular risk assessment and management support. The importance of role clarity and a better understanding of one’s scope of practice, particularly within the context of role and/or service transition, has also been stressed by previous research [33, 34] as critical not just for the management of expectations and hence crucial for successful inter-professional collaboration but also, in turn, for good patient care.

Lastly, participants expressed their strong commitment relating to a positive patient experience and maintaining screening programme standards which could both be undermined through incorporating additional screening within their existing time and resource constrained context. Hence, as highlighted elsewhere with respect to other initiatives and programmes such as the NHS Health Check [35–38], this study’s findings reinforce the importance of adequate resources in terms of funding, staffing and sufficient time made available to staff when introducing additional procedures or responsibilities within an existing service.

Overall our findings reveal multiple challenges along with possible opportunities, as identified by AAA screening programme staff, linked with the proposed multi-component cardiovascular health screening programme. A key issue that follows is regarding the complexities surrounding who may be best placed to implement such a programme, and whether the drive for such a programme needs to come from within or outside of general practice, considering that cardiovascular risk management typically falls within the remit of general practice. The appetite among screening programme staff with respect to the development of their roles is clear from the data, along with an expectation that the proposed programme could potentially offer important benefits for the AAA screening programmes. At the same time, however, the data also reveal some ambivalence among programme staff with respect to the boundaries of their respective roles vis a vis GPs, as well as their own access to adequate resources and systems for the effective performance of their proposed role. In an existing context of low uptake of the NHS Health Check, which is primarily provided through general practice [3, 28, 29], and also increasing workload pressures within UK general practice [34, 39], the NHS AAA screening programmes could potentially offer a more pragmatic pathway for PAD and high BP screening. The feasibility, acceptability, effectiveness and cost-effectiveness of this proposed pathway will be tested within the next stages of the PHAST programme, which will also involve further qualitative interviews with not just the AAA screening programme staff but also with screened patients and GPs to explore their experiences and perspectives.

This study was completed as one of the earlier parts of the larger PHAST programme of work, with the intention that the findings inform, and are followed through into, the later stages of the programme. In this respect, the participants’ responses are based mainly on what they anticipate as the likely opportunities and/or challenges arising from the introduction of the additional screening within their existing work contexts. These views provide a useful insight into the perspectives, concerns and priorities of screening staff – and importantly an understanding of the current AAA screening context, together with staff readiness and support needs in relation to the change. A key strength of this study is the inclusion of participants representing a range of AAA screening sites across England, including a spread of both urban and rural locations and diverse local programme circumstances. We acknowledge that data collection took place within a Corona Virus Disease (COVID) related context of greater time pressures on screening services with respect to addressing backlogs in service provision that may have influenced participants’ views. We also recognise the possible limitation of undertaking telephone follow-on interviews, but suggest that some good initial rapport between the PHAST study researcher and the study participants had already been developed over the course of the one-day face-to-face workshops and focus groups which usefully contributed to the quality of the data collected through the telephone interviews.

## Conclusions

Programme staff simultaneously identified both opportunities and potential challenges linked to the proposed addition of PAD and high BP screening to existing AAA screening programmes. Their views provide useful insights regarding staff concerns and priorities relating to the proposed screening additions within the current AAA screening context. While the additional screening offers potential benefits, the specifics around how this is introduced in practice requires taking into consideration the challenges highlighted by programme staff.

## Data Availability

The dataset arising from this study is not publicly available, as this could compromise participant anonymity and/or the terms of participant consent. Requests for more information may be directed towards the corresponding author.

## Abbreviations

AAA: Abdominal aortic aneurysm
BP: Blood pressure
COVID: Corona virus disease
CVD: Cardiovascular disease
GP: General Practitioner
NHS: National Health Service
NIHR: National Institute for Health Research
PAD: Peripheral arterial disease
PHAST: Peripheral arterial disease, High blood pressure and Aneurysm Screening Trial
UK: United Kingdom
