# Correction: Introducing multi-component cardiovascular health screening into existing Abdominal Aortic Aneurysm (AAA) screening programmes in the UK: a qualitative study of programme staff views

**DOI:** 10.1186/s12913-022-08157-1

**Published:** 2022-06-13

**Authors:** Maria Zubair, Matthew J. Bown, Natalie Armstrong

**Affiliations:** 1 Department of Health Sciences, George Davies Centre, University of Leicester, University Road, Leicester, LE1 7RH UK; 2 Department of Cardiovascular Sciences and NIHR Leicester Biomedical Research Centre, University of Leicester, University Road, Leicester, LE1 7RH UK

**Correction: BMC Health Serv Res 22, 569 (2022)**

**https://doi.org/10.1186/s12913-022-07975-7**

Following publication of the original article [1], the authors identified an error in the formatting of Tables 1, 2 and 3 due to a typesetting mistake. The correctly formatted tables are given below and the original article has been corrected.

**Table 1:** Subthemes of theme I, with illustrative quotes

Theme I – Perceptions of patient experience and health-related outcomes	
Sub-themes	Quotes
1a) An opportunity to improve health-related outcomes	*“I think it's the fact that, you know, we're attracting essentially every sixty-five-year-old gentleman nationally, aren't we, so – it's almost like a gift ….. to pick up something else is got to be good ......”* (AAA Screening Programme staff, Focus group 7) *“….. people might actually be more inclined to have it done as part of AAA screening than going for like a health check at the GP surgery ….. and it's just about preventing people um from, you know, coming in as an emergency or being referred into the hospital setting ….. until they need it really .....”* (AAA Screening Programme manager & formerly screening technician, Interview 7)
1b) Positive health outcomes dependent on adequate follow-up	“*Well depending on um if there is a (unclear) for the GP to act on, or a protocol to act on, then you can expect a bit of a favourable action. Sometimes you do all this – send it to them, and it's ignored or neglected, then it seems pointless, unless it's something to be actioned, or if they have a guideline to say that if this is a possibility, or this is this, this is what we're supposed to do, then we can expect a bit of a benefit or a good outcome of our screening ......*” (AAA specialist nurse & screening technician, Interview 5) *“….. I don't think that every man found to have PAD will go and book an appointment with their GP ….. I think it would be more beneficial for the patient to be booked an appointment – even if it was just a one-off, with the vascular nurse ….. They would be able to explain to them exactly why it's important they make the changes that they do make ….. because some GP surgeries would be better than others if the man does even attend …..*” (AAA Screening Programme manager & formerly screening technician, Interview 7)
1c) PAD+BP+AAA screening’s impact on patient experience	*“And I see it as the more you're doing for one quick appointment ….. they don't have to come back again ..... they're only coming one appointment, they're not having two appointments, even if it's twenty minutes or half an hour – it's not like they'll have to be there for three hours, and have to pay like loads for the parking .....”* (AAA Screening Programme staff, Focus group 6) *“I’m on about to make the appointment as efficient as possible for the patient – we need something that you just literally clip on the toe or clip on the arm – or put on the arm , and there you go – there's your result ..... The easier things are to administer, you don't have the stresses and anxieties of um you know – difficulties being experienced while you're undertaking those tests …..”* (AAA Screening Programme staff, Focus group 8)

**Table 2:** Subthemes of theme II, with illustrative quotes

Theme II – Opportunities and challenges for programme staff	
Sub-themes	Quotes
2a) Positive extension and growth in screeners’ role	*“….. it is a role that doesn't go anywhere ….. With the introduction of this health screen, it has transferability into other screening programmes ….. like I said, it isn't a role where they can extend upwards, past the one before ..... as this will be an additional element in their role that they'll be screening two conditions um rather than one ..... so I think overall – yes, it is a positive thing. With the detection rate for triple A is declining, I think there needs to be an additional um function to the role as well – to make the – to ensure the continued viability of the screening programme …..”* (AAA Screening Programme administration manager, Interview 1) *“So, in some programmes, technicians become bored because of the repetitiveness of the triple A, and if you introduce um PAD and BP screening, then that will introduce different element to their role .....”* (AAA Screening Programme staff, Focus group 8)
2b) Incorporating additional procedures in a resource-constrained context	*“….. additional time to remove extra clothing ….. I mean at the moment all we do is, we just ask gentlemen to lift their upper clothes up – that's all we have to do, so if you're incorporating ‘You need to remove your shoes and socks, then you know, that's a whole different – a whole different game.”* (AAA Screening Programme staff, Focus group 6) *"I think a lot of our screening programmes did ten minutes with two technicians – that obviously increased to initial Covid-secure, sort of, um appointment. Um so it's gonna be interesting ….. will be interesting to see if programmes can actually do that.”* (AAA Screening Programme staff, Focus group 5) *"….. in how we work – it would almost, if we were doing the trial – that it would almost be better to have a separate clinic, specifically for that, so it wouldn't – ‘cause obviously we've all got catch-up to do after Covid, you know – we're way behind in our numbers. So like, in the next few years, you know – we've got pressure already there from – from – ‘cause we've got 9,000 people to scan a year .....”* (AAA Screening Programme staff, Focus group 2)
2c) Reconfiguration of roles, responsibilities and relationships	*“You’re there to screen, and the results ….. I think as well, when it comes to giving results ….. because, you know, managing the blood pressure is really primary care, isn’t it. It’s down to the GP. Um you know, how much do you say to these people ..... It has to be very clearly defined ..... it's quite clear (within AAA screeening programme), isn't it – you're also um getting them an appointment with the nurse. You are actually responsible for the next stages with the nurse, whereas with this (i.e. PAD+BP screening) ..... you're sending them up to the GP, which is really mixed – everything is different .....”* (AAA Screening Programme staff, Focus group 2) *“The other thing would be the actual – because it's GP that decides whether they – what they decide to do or not ..... With the aneurysm screening ..... two weeks on, or whatever the timescales are, you’re likely to have an operation – not definitely – not guaranteed, but it's a possibility. Actually, if you go to GP, he's too busy to do – or doesn't think the patient has got peripheral arterial disease, or something like that – very – quite variable.”* (AAA Screening Programme staff, Focus group 5)

**Table 3:** Subthemes of theme III, with illustrative quotes

Theme III – Maintaining and improving programme standards	
Sub-themes	Quotes
3a) Maintaining AAA programme standards	*“As long as you've got time for it – not to impact on the quality of the triple A screening ..... We've got it (i.e. AAA screening) good, and how we do that, and you know – and the timing we've got, you know, is appropriate for that …..”* (AAA Screening Programme staff, Focus group 2) *“It's always standards, isn't it – you tend to sort of set up your achievable and your acceptable standards – your thresholds um – how they're gonna be monitored. Um I think it is your appointment times, um practicalities, equipment – buying the equipment, whose gonna buy the equipment – whose going to actually um replace the equipment .....”* (AAA Screening Programme staff, Focus group 5) *“If you've got lots of false positives or false negatives then it undermines the whole screening programme ..... so you'd want to know who would be looking at what we're doing, and whether or not it's actually being checked to make sure ..... we need a QA (Quality Assurance) .....”* (AAA Screening Programme staff, Focus group 6)
3b) An opportunity to improve service-delivery	*“Could this be a way of getting that letter into – via what's called the Patient Knows Best app, so you can opt into it so you get copies of letters that go to your GP that's about you – whereas the triple A isn't in there – whereas if you're getting funding, if you've got this then the patient could have the letter ….. I mean, you give them the result on the day, but a lot of the time they don't take in a verbal result ….. For certain people, they like to have written results ..... So if you're thinking of electronically doing a lot of stuff, then maybe we can do this .....”* (AAA Screening Programme staff, Focus group 6) *“I think it would benefit us ….. it's an incentive for the GPs – whereas GPs I've found are very dismissive of us when we go. I mean, we do pay for the room, but we're more of an inconvenience at the minute – whereas if GPs are on board with this, and we're doing the service in there ….. So hopefully they'll play their part a bit better than what they do now ….. I think they're gonna be more on board with it and they'll probably welcome us to come around – ‘cause also then, we're doing it, they're not having to train up a lot of staff to do it for them ….. it will open, I think, more doors for us ….. I think, this will benefit triple A as well ..... we feel like we’re in the way a little bit although we try not to ..... we just think this will be an incentive for them.”* (AAA Screening Programme staff, Focus group 6)
